# Supplementary material for: Beyond Bonferroni: new multiple contrast tests for time-to-event data under non-proportional hazards
Source: Lifetime Data Anal. 2026 Jan 14;32(1):8. doi: 10.1007/s10985-025-09676-9 (PMC12804333; doi:10.1007/s10985-025-09676-9)
Supplement: Supplementary file 1 — (pdf 178 KB) [file 10985_2025_9676_MOESM1_ESM.pdf]

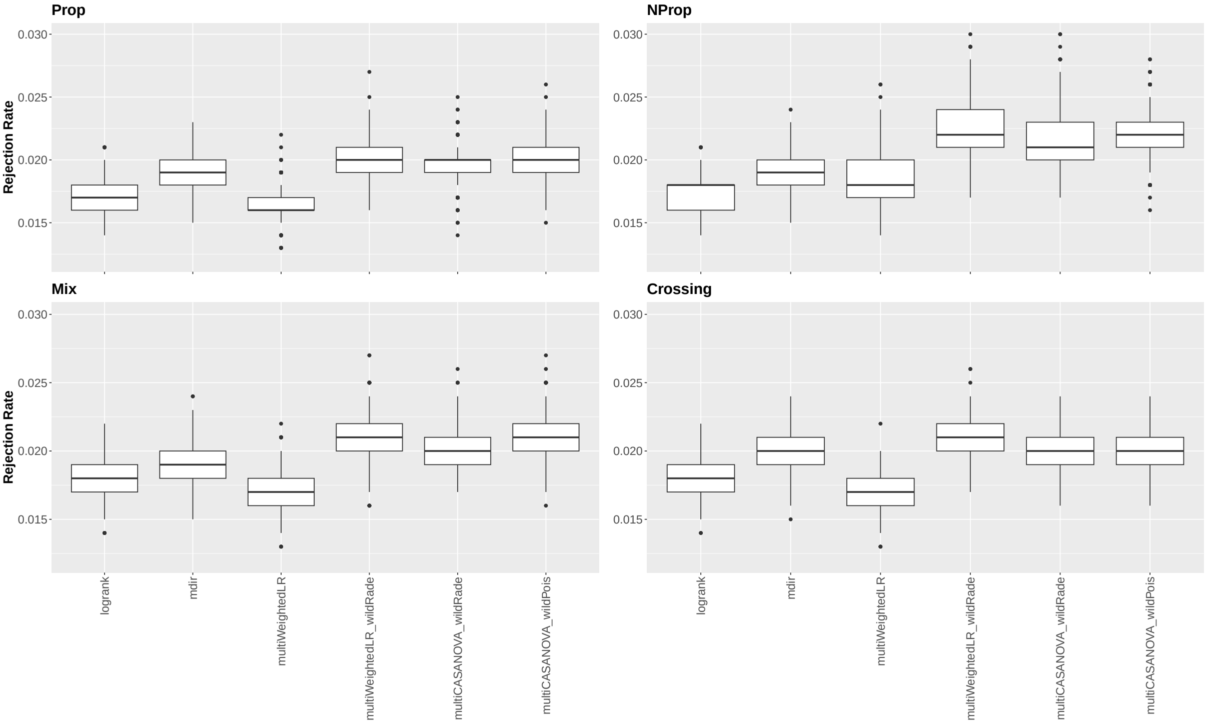

Figure S1: Rejection rate of the local tests with no difference in survival for the Dunnett-type matrix.

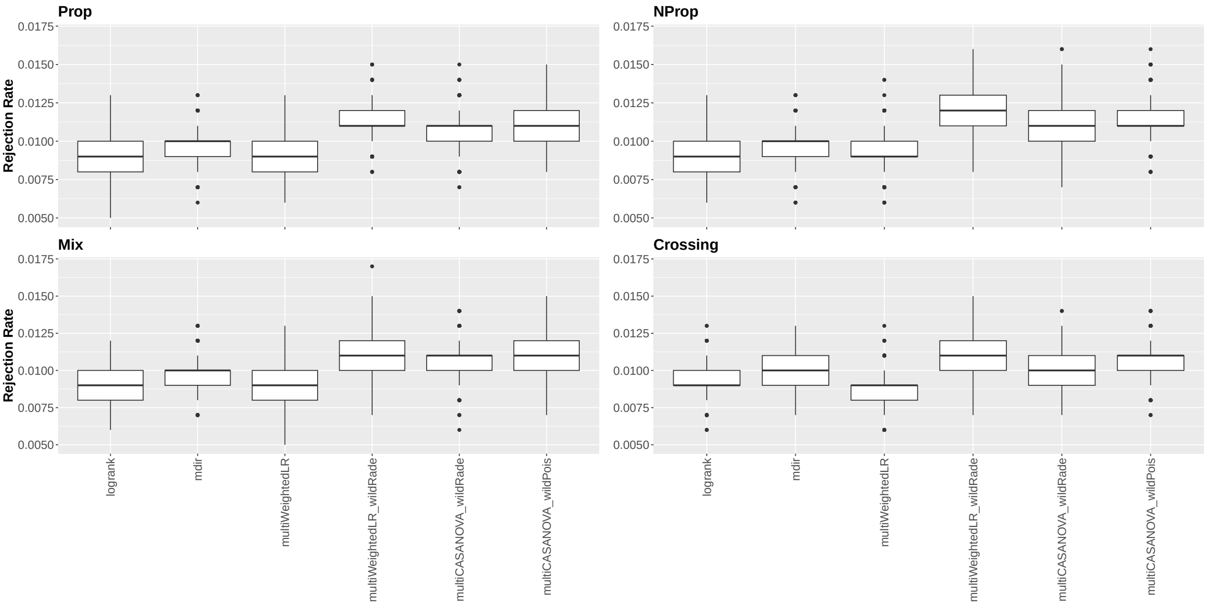

Figure S2: Rejection rate of the local tests with no difference in survival for the Tukey-type matrix.

Table S1: p-values for the local comparisons in the data example (Section 4). Significant tests to level α or $\alpha_{\text{Bonferroni}}$ are highlighted in bold font.

	log-rank	mdir	multiWeightedLR	multiCASANOVA_wildRade	multiCASANOVA_wildPois
2 - 1	0.196	0.411	0.130	0.301	0.304
3 - 1	0.100	0.152	0.415	0.699	0.727
4 - 1	**0.001**	**0.003**	**0.013**	**0.030**	**0.023**
5 - 1	**0.010**	**0.028**	0.135	0.203	0.210
6 - 1	0.502	0.515	0.981	1.000	1.000
7 - 1	0.040	0.136	0.366	0.571	0.566
3 - 2	**<0.001**	**0.001**	**0.006**	**0.012**	**0.005**
4 - 2	**<0.001**	**<0.001**	**0.001**	**0.003**	**<0.001**
5 - 2	**<0.001**	**<0.001**	**0.003**	**0.007**	**0.001**
6 - 2	**0.002**	**0.001**	**0.040**	0.092	**0.091**
7 - 2	**<0.001**	**<0.001**	**0.006**	**0.011**	**0.003**
4 - 3	0.406	0.217	0.821	0.980	0.991
5 - 3	0.612	0.759	0.999	1.000	1.000
6 - 3	0.242	0.534	0.929	0.985	0.995
7 - 3	0.889	0.943	1.000	1.000	1.000
5 - 4	0.885	0.990	0.956	0.981	0.993
6 - 4	0.034	0.046	0.175	0.415	0.414
7 - 4	0.777	0.768	0.760	0.922	0.942
6 - 5	0.195	0.329	0.649	0.776	0.789
7 - 5	0.959	1.000	0.999	1.000	1.000
7 - 6	0.424	0.248	0.907	0.941	0.959

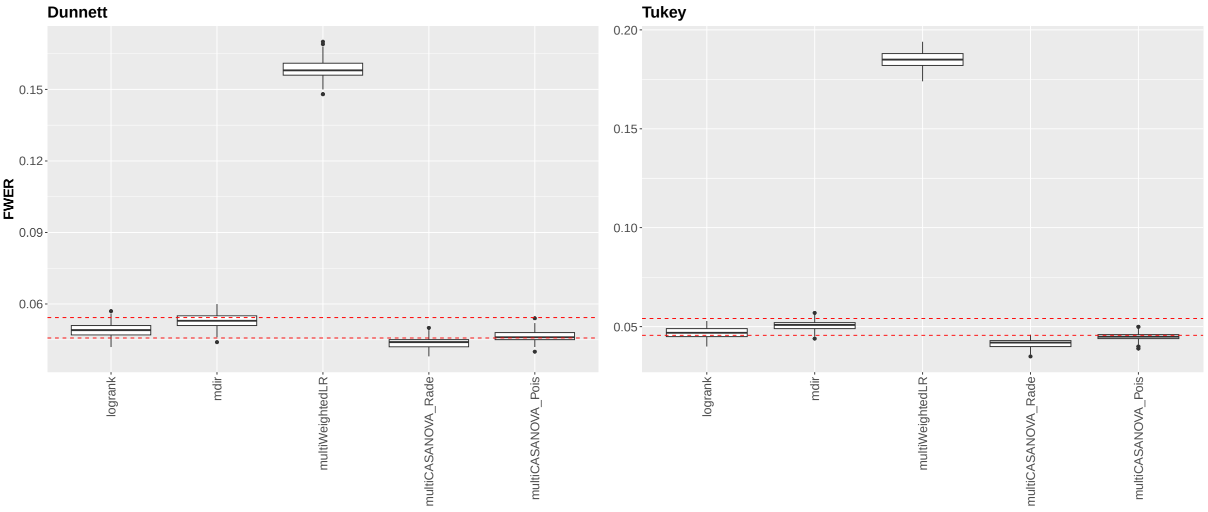

Figure S3: FWER under $\mathcal{H}_0$ for all settings for the Dunnett-type (left) and Tukey-type (right) contrast matrices for $n = 50$. The dashed lines represent the borders of the binomial confidence interval $[4.57\%, 5.43\%]$

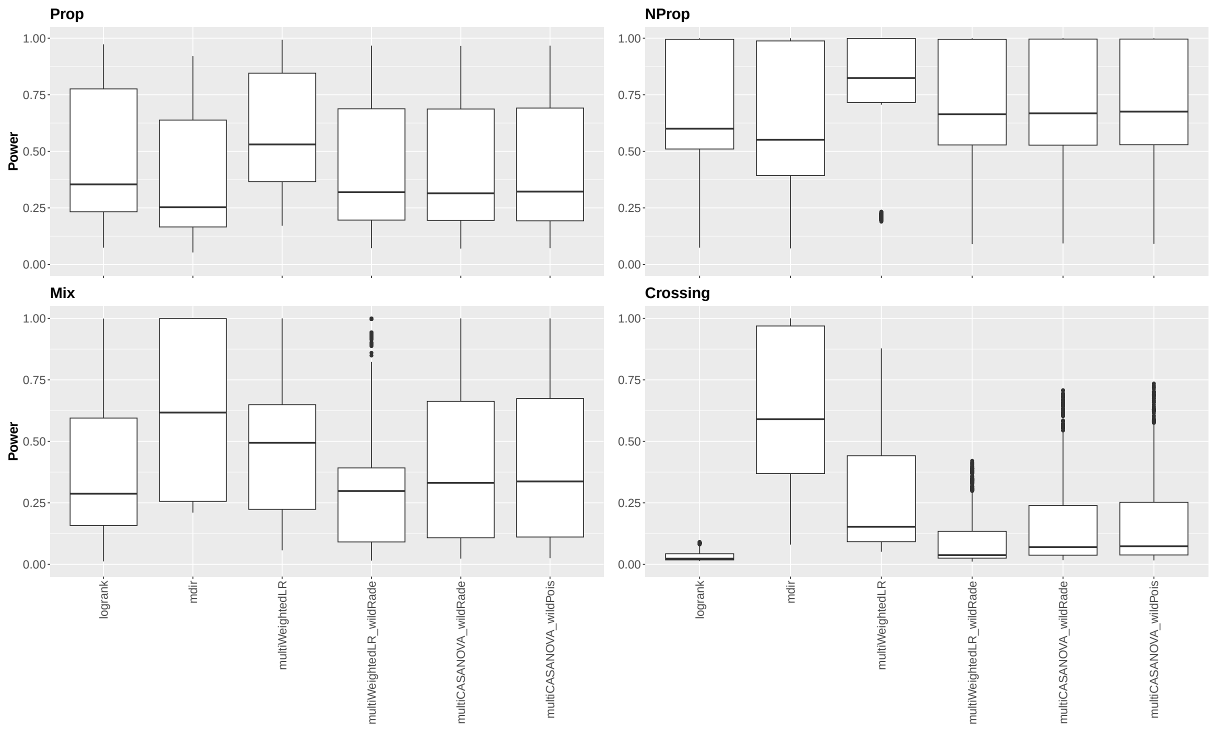

Figure S4: Local power over all tests under the alternative for $n = 50$ for Dunnett-type contrasts for all four scenarios (each boxplot contains 1136 data points).

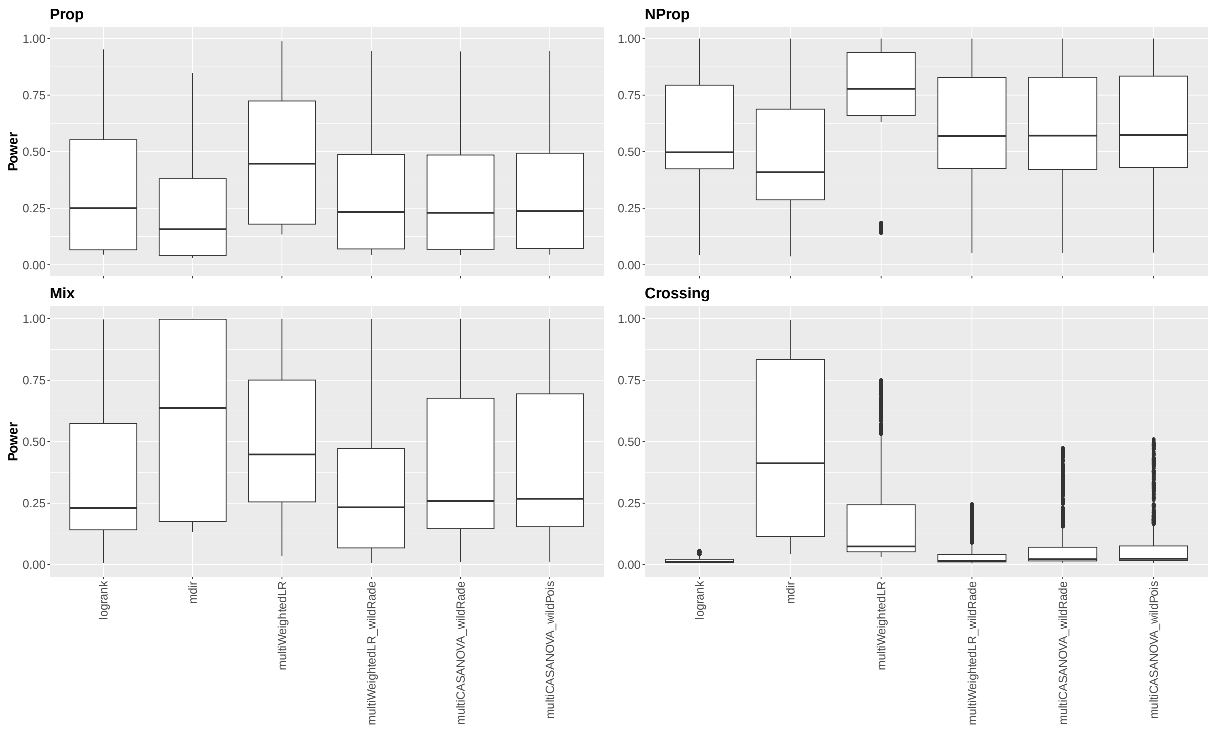

Figure S5: Local power over all tests under the alternative for $n = 50$ for Tukey-type contrasts for all four scenarios (each boxplot contains 2016 data points).
